# Effect of modification of peanut conglutin on IgE and T cell reactivity in adults with peanut allergy

**DOI:** 10.1186/2045-7022-1-S1-O16

**Published:** 2011-08-12

**Authors:** Els van Hoffen, Hanneke van der Kleij, Stans den Hartog Jager, Edward Knol, Dirk-Jan Opstelten, Stef Koppelman, André Knulst

**Affiliations:** 1 University Medical Center Utrecht, dep. Dermatology/Allergology, Utrecht, Netherlands; 2 HAL Allergy BV, Leiden, Netherlands

## Background

In peanut-allergic patients, specific immunotherapy using peanut extract has been shown to be associated with significant side effects. Chemically modified allergens may provide a safer alternative.

## Methods

Purified native peanut conglutin, containing Ara h2 and Ara h6, was chemically modified by reduction and alkylation (RA), without or with additional glutaraldehyde treatment (RAGA). Serum of 14 peanut allergic patients (DBPCFC-confirmed) was analyzed for peanut-specific antibodies by IgE immunoblot and IgE/IgG1/IgG4 ELISA. Allergen-induced basophil activation was analyzed by direct basophil activation test (BAT), using whole blood and washed whole blood to remove the plasma. Native conglutin-specific T cell lines were analyzed for proliferation and cytokine production.

## Results

IgE binding on immunoblot and IgE, IgG1 and IgG4 binding by ELISA to RA and RAGA was significantly reduced compared to native conglutin. In most patients, whole blood basophil activation by RA and RAGA was reduced compared to native conglutin. In 4 patients, modification did not result in a reduced basophil response. These 4 patients had significantly higher levels of native conglutin-specific IgG1 (ELISA) compared to the other 10 patients. Upon washing of whole blood, the response to native protein increased. The response to RA and RAGA remained unchanged and was now reduced compared to native conglutin in all patients. T cell proliferation to RA was unchanged, and to RAGA reduced as compared to native conglutin. Levels of cytokines (IL-13, IFN-g, TNF-a) correlated with proliferation. No IL-10 production was observed.

## Conclusions

Modification of peanut conglutin overall reduces IgE/IgG binding. Allergen-induced basophil activation is reduced in most patients. In some patients, the whole blood basophil response to RA and RAGA is relatively stronger than to native conglutin, which may be due to inhibitory IgG1 antibodies that suppress the response to native conglutin. T cell immunogenicity is retained, especially to RA. This shows that chemical modification may be a promising approach for the development of immunotherapy for peanut allergy.

